# Supplementary material for: WordCommentsAnalyzer: A windows software tool for qualitative research
Source: F1000Res. 2018 Sep 4;7:536. Originally published 2018 May 3. [Version 2] doi: 10.12688/f1000research.14819.2 (PMC6137411; doi:10.12688/f1000research.14819.2)
Supplement: Supplementary file 3 [file f1000research-7-17614-s0002.tgz › b2a79496-4ada-47c4-9682-a99ce867836d.zip!/Eight Iranian Health Journals/Journal of Research in Rehabilitation Sciences _49.docx]

© Journal of Research in Rehabilitation Sciences . This work is licensed under CC BY-NC 4.0 (https://creativecommons.org/licenses/by-nc/4.0/).

Volume & Issue: دوره 5، شماره 1: 1388

Title: تاثیر برنامه های Back School بر کاهش میزان کمردرد و تغییرات قوسهای ستون فقرات

http://jrrs.mui.ac.ir/index.php/jrrs/article/view/32

مقدمه: اغلب تحقیقات نشان داده‌اند که دستور‌العمل‌های BACK SCHOOL روش مؤثری جهت افزایش سطح دانش کارگران است. هدف پژوهش حاضر بررسی تأثیر برنامه‌های  Back Schoolبر کاهش میزان کمردرد و تغییرات قوس‌های ستون فقرات می‌باشد. مواد و روشها: در این پژوهش کارگران کارخانه ایران خودرو، از سه شرایط کاری مختلف (300نفر) با میانگین سنی6/6 ± 31 سال انتخاب شدند. ابتدا زاویه لوردوز کمری، کایفوز سینه‌ای و میزان کمردرد آن‌ها اندازه‌گیری شده و سپس به دو گروه تقسیم‌بندی شدند و برنامه‌های Back School روی یکی از گروه‌ها اجرا گردید. آزمون آماری تی مستقل و تی زوجی برای تجزیه و تحلیل آماری مورد استفاده قرار گرفتند. یافته‌ها: نتایج آزمون آماری تی زوجی نشان داد که در گروه اجرا کننده برنامه‌های Back  Schoolتغییر قابل توجهی در زاویه لوردوز کمری (p = 0.672)، کایفوز سینه‌ای (p = 0.517) و میزان کمردرد (p = 0.399) آزمودنی‌ها مشاهده نشد. بحث: بر اساس نتایج تحقیق حاضر نتیجه‌گیری می‌شود که شرایط کاری عامل اثر گذاری روی زاویه لوردوز کمری، کایفوز سینه‌ای و میزان کمردرد افراد می‌باشد و برنامه‌های Back School یکی از بهترین برنامه‌ها برای حفظ وضعیت مطلوب در شرایط کاری است.

Title: بررسی تأثير روش‌هاي مختلف حمل كوله‌ پشتي بر حجم‌هاي تنفسي در دانشجويان دختر

http://jrrs.mui.ac.ir/index.php/jrrs/article/view/33

مقدمه: مطالعات گسترده‌ای در مورد تأثیرات حمل بر وضعیت و سیستم عضلانی اسکلتی انجام شده است، اما در مورد اثرات حمل کوله‌ پشتی بر عملکرد ریوی مطالعات کمی وجود دارد. هدف از این پژوهش بررسی اثر روش‌های مختلف حمل با 15 درصد وزن بدن بر روی حجم‌های تنفسی و مقایسه آن‌ها با یکدیگر بود. مواد و روش‌ها: در این مطالعه تعداد 70 نفر فرد سالم با دامنه سنی 25-18سال و میانگین 55/1 ± 47/20 سال شرکت کردند. حجم‌های تنفسی ابتدا در حالت بدون کوله ‌پشتی ارزیابی شد. سپس کوله ‌پشتی در یکی از حالت‌های حمل یک‌طرفه، مورب و دو طرفه روی بدن قرار گرفت و دو‌باره حجم‌های تنفسی اندازه‌گیری شد. وسیله مورد استفاده جهت اندازه‌گیری حجم‌های تنفسی دستگاه اسپیرومتر Oxycon بود. آنالیز آماری توسط آزمون مقایسه زوج‌ها (Paired t-test) انجام شد. یافته‌ها: مقایسه مقادیر FEV1، FVC و درصد FEV1/FVC در حالت بدون کوله‌ پشتی با سه روش حمل یک‌‌طرفه، مورب و دو طرفه تغییرات معنی‌داری نشان نداد و در مقایسه این مقادیر در سه روش حمل یک‌‌طرفه، مورب و دو طرفه به صورت دو به دو نیز تفاوت معنی‌داری مشاهده نشد. بحث: حمل کوله ‌پشتی با 15 درصد وزن بدن به سه شکل یک‌طرفه، مورب و دو طرفه تغییر معنی‌داری در حجم‌های تنفسی ایجاد نکرد و بین روش‌های حمل نیز تفاوت معنی‌داری دیده نشد. بنابراین، به نظر نمی‌رسد که حمل بار با 15 درصد وزن بدن بر روی سیستم تنفسی اثرات نگران‌کننده‌ای داشته باشد.

Title: قابلیت وضوح گفتار در کودکان 4 تا 5 سال فارسي زبان

http://jrrs.mui.ac.ir/index.php/jrrs/article/view/34

مقدمه: قابلیت وضوح گفتار، اندازه‌‌گیری میزان مؤثر بودن گفتار است. قابلیت وضوح گفتار در سه زمینه: تصمیم‌‌‌گیری برای مداخله، تعیین اهداف مداخله، ارزیابی تأثیر مداخله کاربرد دارد. هدف از این مطالعه، بررسی قابلیت وضوح گفتار در کودکان 4 تا 5 سال بود. مواد و روش‌ها: پژوهش حاضر، یک مطالعه توصیفی- مقطعی بود که 70 کودک ( 35 پسر و 35 دختر ) به صورت تصادفی ساده از میان پنج  ناحیه شهر اصفهان به عنوان نمونه انتخاب شدند، به طوری که از هر ناحیه دو کودکستان و از هر کودکستان نمونه گفتاری، هفت کودک به منظور تعیین قابلیت وضوح گفتار مورد بررسی قرار گرفت. یافته‌ها: میانگین درصد قابلیت وضوح گفتار، در کل جمعیت نمونه 97/53 ( 2/47 = SD ) بود، دامنه آن برابر 90 تا 100 است. میانگین درصد قابلیت وضوح گفتار در جمعیت پسران 98 (2/7SD = ) و در جمعیت دختران 97/6 (2/9SD = ) بود. آزمون t مستقل نشان داد كه تفاوت ميانگين دختران و پسران معني‌دار نمي‌باشد (074/0 = P). بحث: بر طبق این نتایج، قابلیت وضوح گفتار در کودکان 4 تا 5 سال، تقربباً مشابه بزرگسالان بود.

Title: ارزيابي فراواني نا هنجاري‌هاي لوردوز و كيفوز در سنين مختلف

http://jrrs.mui.ac.ir/index.php/jrrs/article/view/35

مقدمه: هدف از تحقيق حاضر، ارزيابي فراواني نا هنجاري‌هاي لوردوز و كيفوز در سه رده سني شهرستان بندرعباس بود. مواد و روش‌ها: 349 نفر در رده‌هاي سني؛ نوجوان (141 نفر)، جوان (114 نفر) و ميان‌سال (94 نفر) به عنوان نمونه تحقيق انتخاب شدند. براي به دست آوردن انحناي ستون فقرات از خط كش منعطف (دقت 1/0 درجه) و جهت به دست آوردن زاويه قوس‌ها از روش Cobb و از آزمون كاي اسكور براي تعيين نسبت در بين گروه‌ها استفاده شد. یافته‌ها: در ارزيابي قوس كمري، نتايج تحقيق نشان داد که تعداد افراد نرمال (1/34 درصد) نسبت به افراد داراي پشت صاف (6/10 درصد)، لوردوز ( 8/25 درصد) و لوردوز شديد (5/29 درصد) بيشتر بود، پس از انجام آزمون آماري تفاوت معنی‌داري بين نمونه‌ها مشاهده شد (05/0P < ). بيشترين نا هنجاري لوردوز و لوردوز شدید به ترتیب زنان جوان و میان‌سال مشاهده شد (8/38 و 7/48 درصد). نتايج تحقيق نشان داد كه از مجموع 349 نفر؛ بيشتر افراد وضعيت نرمال داشتند (3/55 درصد)، بين نسبت‌هاي افراد پشت صاف (4/3درصد)، داراي كيفوز (2/27 درصد) و كيفوز شديد (1/14 درصد) تفاوت معنی‌داري مشاهده شد (05/0P < ). تفاوت معنی‌داري در ميزان كيفوز بين نوجوانان (85/29 درصد)، جوانان (85/29 درصد) و ميان‌سالان (3/40 درصد) مشاهده نشد (05/0P > ). مردان ميان‌سال داراي بيشترين ميزان فراواني نا هنجاري كيفوز و کیفوز شدید بودند (9/31 درصد) كه تفاوت معنی‌داري با ديگر گروه‌ها داشت (05/0 > P). بحث: در کشورهاي پيشرفته از طريق تست‌هاي مختلف، از همان دوران اوليه افراد مختلف را در رده‌هاي سني گوناگون مورد ارزيابي و تست نا هنجاري‌هاي بدني قرار مي‌دهند و پيش‌گيري از پيدايش چنين نا هنجاري‌هايي را از سنين اوليه زندگي آغاز مي‌نمايند. بنابراين، ضرورت انجام چنين تست‌هايي در کليه سطوح تحصيلي اعم از دبستان، راهنمايي، دبيرستان، دانشگاه‌ها و حتي اساتيد دانشگاه‌ها وجود دارد تا نسبت به پيشگيري از بروز چنين ناهنجاري‌هايي در سنین پیری و هم‌چنين درمان آن اقدام لازم صورت گيرد.

Title: تعيين ميزان مطابقت ارجاع بيماران مبتلا به علايم درد سياتيکي به جراحي با شاخص‌هاي استاندارد درماني

http://jrrs.mui.ac.ir/index.php/jrrs/article/view/36

مقدمه: کمردرد، اولين علت ناتواني در افراد زير 45 سال و دومين علت مراجعه به پزشکان است. بيماران، با تشخيص قطعي فتق ديسک بايد طي 8 هفته تحت درمان‌هاي غير جراحي قرار گيرند، مگر در مواردي که فرد ضعف پيش رونده حرکتي، سندرم دم اسبي و يا درد غير قابل مهار داشته باشد. تنها 10-5 درصد بيماران با علايم درگيري ديسک بين مهره‌اي نياز به جراحي دارند. هدف اين مطالعه، تعيين ميزان تطابق ارجاع بيماران مبتلا به فتق ديسک کمري به جراحي با توجه به شاخص‌هاي استاندارد بود. مواد و روش‌ها: اين مطالعه توصيفي و متقاطع (cross-Sectional)، روي 46 بيمار با ميانگين سني 40 سال انجام شد. از هر بيمار رضايت‌نامه کتبي گرفته شد. سپس هر بيمار به سؤالات مربوط به مشخصات درد و شاخص‌هاي جراحي پاسخ داد و شدت درد به وسيله VAS و شدت سياتيکا توسط SLR اندازه‌گيري شد. اين نتايج به همراه اطلاعات به دست آمده از برگه گزارش MRI ثبت گرديد و اطلاعات جمع‌آوري شده براي تحليل با روش‌هاي آمار توصيفي، وارد برنامه SPSS شد. یافته‌‌ها: تعداد 13 درصد بيماران، فاقد درد راديکولار بودند و در 85 درصد آن‌ها، از شروع درد راديکولار کمتر از 6 ماه مي‌گذشت. درمان‌هايي که اين 46 بيمار دريافت کرده بودند به ترتيب دارو درماني (85 درصد)، استراحت (65 درصد)، کمربند طبي (30 درصد) و فيزيوتراپي (22 درصد) بود. مؤثرترين درمان کانسرواتيو، کمربند طبي با تأثیر 29 درصد بود. نتيجه‌گيري: بسياري از بيماران اين مطالعه، به صورت غير ضروري جراحي شده‌اند؛ چرا که شاخصه‌هاي جراحي را نداشته و درمان کانسرواتيو مناسبي دريافت نکرده بودند. نتايج درمان کانسرواتيو اين بيماران به خصوص در مورد فيزيوتراپي ضعيف است، اگر چه در مقالات به نقش زياد اين درمان تأکيد شده است.

Title: رشد فهرست آوايي درکودکان فارسی زبان 6-2 ساله

http://jrrs.mui.ac.ir/index.php/jrrs/article/view/37

مقدمه: کودکان درپایان اولین سال زندگی خود اولین واژه هارا تولید می کنند. با افزایش سن تعداد واژه ها و تعداد واج هادرفهرست آوایی کودک افزایش می یابد. اولین همخوان ها درفهرست آوایی کودکان همخوان های خیشومی، انفجاری، سایشی، لبی و زبانی می باشند. با افزایش سن، کودکان از همخوان های بیشتری درگفتارخود استفاده می نمایند.آسیب شناسان گفتار و زبان جهت قضاوت در مورد طبیعی یا غیر طبیعی بودن گفتارکودکان به اطلاعات حاصل ازمطالعات جنبه های مختلف گفتاردردوران رشد نیازمند می باشند. هدف ازاین مطالعه تعیین سن اکتساب همخوان ها درکودکان فارسی زبان است. مواد و روش‌ها: پژوهش حاضر يك مطالعه توصيفي- تحليلي و مقطعي است. نمونه گفتار 96 کودک 6-2 ساله طبیعی فارسی زبان شهرتهران مورد مطالعه قرار گرفت. با استفاده از44 تصوير، چگونگی تولید 22 همخوان فارسی درموقعیت آغازین وپایانی واژه های یک و دوسیلابی مورد بررسی بود. یافته ها: همخوان های دولبی خیشومی وانسدادی/b/,/p/,/m/ مقدم برسایرهمخوان ها توسط کودکان 5/2-2 ساله دردوموقعیت واژه صحیح تولید شدند.همخوان های سایشی و سایشی انسدادی /ž/,/ĵ/ دیرتر از سایر همخوان ها دردو موقعیت واژه توسط کودکان مورد پژوهش بعد از 4 سالگی صحیح تولیدشدند.آزمون رگرسیون این فرضیه را اثبات کرد که با افزایش سن تعداد خطاهای تولید همخوان ها توسط کودکان کاهش یافت. نتيجه‌گيري: کودکان فارسی زبان تا پایان 4 سالگی می توانند همخوان ها را به غیر از /ž/,/ĵ/ در قالب واژه های یک و دو سیلابی درموقعیت آغازین و پایانی صحیح تولید نمایند.این نتیجه با نتایج پژوهش های مشابه درزبان انگلیسی هم سو می باشد.واج های سایشی در زبان انگلیسی نیزسخت ترازسایر واج ها می باشند.

Title: تجارب خانواده‌های دارای كودك معلول: یک مطالعه پدیدارشناسی

http://jrrs.mui.ac.ir/index.php/jrrs/article/view/38

مقدمه: تولد كودك معلول حادثه‌‌ای است كه بر تمام اعضا خانواده و ارتباطات داخلی و خارجی آن اثر می‌گذارد و نیاز به بررسی عملكرد خانواده دارد. شواهد نشان می‌دهد كه بعضی از خانواده‌ها هرگز به طور كامل نسبت به این حادثه تطابق پیدا نمی‌كنند. از آن جا که شناخت تجارب خانواده‌های دارای فرزند معلول، مددكار را به شناخت عمیقی از وضعیت خانوادگی، روابط فردی و اجتماعی این افراد می‌‌رساند نتایج این تحقیق می‌تواند كمك كننده باشد. مواد و روش‌ها: موضوع مورد مطالعه در این تحقیق، تجارب خانواده‌های دارای كودك معلول بود و با توجه به این كه پدیده مورد مطالعه در این پژوهش، تجارب انسانی بود، از روش پدیده شناسی استفاده شد. نمونه‌گیری مبتنی بر هدف بوده و به منظور رسیدن به اهداف پژوهش با 12 نفر از اعضای خانواده‌هایی که دارای کودک معلول بودند و کودک آن‌ها در مراكز نگهداری معلولین ذهنی اداره بهزیستی نگهداری می‌شد مصاحبه شد. پژوهشگر با مصاحبه و مكالمه عمیق و سازمان نیافته كه بر طرح سؤالات وسیع، باز و محوری استوار بود به شركت كننده كمك نمود تا تجارب زندگی خود را بیان كند. در این پژوهش روش تجزیه و تحلیل داده‌ها براساس روش هفت مرحله‌ای كلایزی انجام شد. یافته‌ها: مصاحبه انجام شده از 12 شرکت کننده روی كاغذ پیاده گردید و سخنان مهم جدا شد و 74 كد استخراج شد.از بین آن‌ها مفاهیم اصلی ایجاد شد. این مفاهیم در دسته ‌بندی‌های موضوعی اصلی قرار داده شدند و به این ترتیب کدهای استخراج شده در پنج مفهوم اصلی به این شرح طبقه‌بندی شدند:1_ساختار حمایتی،2_ابزارهای تطابقی،3_تعاملات، 4_مشخصه‌های کودک، 5_بار روحی روانی. بحث: نتایج تحقیق نشان می‌دهد وجود کودک معلول منجر به مشکلات روحی روانی و تغییرات وسیعی در کلیه ابعاد زندگی کلیه اعضا خانواده می‌شود و بر تعاملاتشان اثر می‌گذارد. اعضا خانواده جهت تطابق خود و سازگاری و یا برگشتن به زندگی عادی راه‌های متفاوتی را در پیش می‌گیرند و با وجود مشکلات روحی روانی و تعارضاتی که پیدا می‌کنند به عنوان یک کودک ضعیف با نا توانی‌های خاص خود و متعلق به آن‌ها درصدد حمایت از او بر می‌آیند. مشکلات روانی، اجتماعی و اقتصادی كه خانواده در حین سازگاری با بچه معلول تجربه می‌كنند می‌تواند منجر به تعارضاتی در خانواده شود و ممكن است منجر به تغییراتی در نقش‌های افراد و عملكرد آن‌ها در خانواده شود.

Title: تدوين ابزاري جهت تشخيص آپراكسي دهاني ((Oral aproaxia در كودكان 4 تا 5 ساله

http://jrrs.mui.ac.ir/index.php/jrrs/article/view/39

مقدمه: حجم قابل ملاحظه‌اي از كودكان با اختلالات توليدي كه جهت درمان به كلينيك‌هاي گفتاردرماني مراجعه مي‌كنند از ديد پاتولوژي (Pathology) هيچ يك از علت‌هاي معمول در ايجاد اختلال توليدي (از قبيل ناهنجاري ساختماني، فقر محيطي، عقب ماندگي ذهني، كم شنوايي و ...) را نشان نمي‌دهند. با توجه به ارتباطات زيادي كه محققين بين اختلالات توليدي و آپراكسي دهاني قایل شده‌اند وجود آپراكسي دهاني در اين مراجعين بسيار محتمل مي‌باشد . بنابراين، براي تشخيص آپراكسي دهاني در اين كودكان به يك ابزار كارآمد نياز است. مواد و روش‌ها : در اين مطالعه توصيفي– تحليلي در ابتدا با استفاده از منابع، يك چك ليست ارزيابي آپراكسي دهاني شامل؛ 20 تكليف تهيه و روايي و پايايي آن مشخص گرديد (2، 1). سپس 74 نمونه عادي در گروه سنی 4 تا 5 سال انتخاب شدند. براي تأیید نرمال بودن نمونه‌‌ها علاوه بر بررسي تاريخچه پزشكي، دو آزمون براي هر كدام انجام گرديد؛ آزمون آوايي، جهت تعيين وضعيت تلفظي نرمال و ديگري آزمون ارزيابي حلقي– دهاني (3)، براي بررسي عدم وجود مشكلات حسي– حركتي در ناحيه دهان و صورت. سپس توانايي انجام تكاليف چك ليست آپراكسي دهاني براي بدست آوردن نقطه برش، جهت تشخيص فرد مبتلا در نمونه‌ها، بررسي شد. تعيين نقطه برش با بررسي ويژگي (Specifity) و مثبت كاذب (False positive) بدست آمده از داده‌ها انجام گرفت. یافته‌ها: براساس يافته‌هاي حاصل از اين پژوهش، 15 تكليف از 20 تكليف چك ليست آپراكسي دهاني به عنوان نقطه برش تشخيص فرد مبتلا در اين محدوده سني تعيين گرديد. با انتخاب 15 تكليف، ميزان ويژگي برابر (6/94 درصد) و مثبت كاذب برابر (4/5 درصد) است. اين مطلب بيانگر آن است كه اگر كودكي 15 تكليف يا كمتر را انجام دهد دچار آپراكسي دهاني است و اگر توانايي انجام بيش از 15 تكليف را دارا باشد، سالم مي‌باشد. بحث: با توجه به مطالعه حاضر، ابزار بدست آمده، تشخيص آپراكسي دهاني را در گروه‌هاي سني خاصي از كودكان هموار مي‌سازد. اگر در كودكان اختلالات توليدي، آپراكسي دهاني وجود داشته باشد، لزوم برنامه‌ريزي و مديريت درمان بر اساس آن از اهميت بالايي برخوردار است.
